# Beta Cell Physiological Dynamics and Dysfunctional Transitions in Response to Islet Inflammation in Obesity and Diabetes

**DOI:** 10.3390/metabo10110452

**Published:** 2020-11-10

**Authors:** Marlon E. Cerf

**Affiliations:** 1 Grants, Innovation and Product Development, South African Medical Research Council, Tygerberg 7505, South Africa; marlon.cerf@mrc.ac.za; 2 Biomedical Research and Innovation Platform, South African Medical Research Council, Tygerberg 7505, South Africa; 3 Division of Medical Physiology, Department of Biomedical Sciences, Faculty of Medicine and Health Sciences, University of Stellenbosch, Tygerberg 7505, South Africa

**Keywords:** beta cell hypofunction, beta cell hyperfunction, ER stress, glucolipotoxicity, oxidative stress

## Abstract

Beta cells adapt their function to respond to fluctuating glucose concentrations and variable insulin demand. The highly specialized beta cells have well-established endoplasmic reticulum to handle their high metabolic load for insulin biosynthesis and secretion. Beta cell endoplasmic reticulum therefore recognize and remove misfolded proteins thereby limiting their accumulation. Beta cells function optimally when they sense glucose and, in response, biosynthesize and secrete sufficient insulin. Overnutrition drives the pathogenesis of obesity and diabetes, with adverse effects on beta cells. The interleukin signaling system maintains beta cell physiology and plays a role in beta cell inflammation. In pre-diabetes and compromised metabolic states such as obesity, insulin resistance, and glucose intolerance, beta cells biosynthesize and secrete more insulin, i.e., hyperfunction. Obesity is entwined with inflammation, characterized by compensatory hyperinsulinemia, for a defined period, to normalize glycemia. However, with chronic hyperglycemia and diabetes, there is a perpetual high demand for insulin, and beta cells become exhausted resulting in insufficient insulin biosynthesis and secretion, i.e., they hypofunction in response to elevated glycemia. Therefore, beta cell hyperfunction progresses to hypofunction, and may progressively worsen towards failure. Preserving beta cell physiology, through healthy nutrition and lifestyles, and therapies that are aligned with beta cell functional transitions, is key for diabetes prevention and management.

## 1. Introduction

Obesity and diabetes are globally pervasive, increasing in prevalence, and occur over the life-course, often presenting earlier in life e.g., in childhood obesity and diabetes. Urbanization, sedentary lifestyles, and unhealthy diets are some drivers of the global high prevalence of obesity and diabetes, with healthy nutrition particularly important for obesity and diabetes prevention and management. In obesity and early diabetes, overnutrition (i.e., hypercaloric overconsumption, excess nutrients, or nutritional overload, such as chronic high-fat diet (HFD)/high saturated fatty acid (SFA) overconsumption), increases the demand for insulin biosynthesis and secretion, leading to the onset of hyperglycemia and insulin resistance. Beta cells are highly specialized to biosynthesize and secrete insulin to maintain glucose homeostasis. Exposure to chronic hyperglycemia (glucotoxicity) and lipids (lipotoxicity) trigger beta cell dysfunction and death [1,2,3], and in combination, i.e., an excess of glucose and lipids (glucolipotoxicity), synergize rapid and progressive beta cell demise [4,5]. Glucolipotoxicity therefore is the combined deleterious consequences of elevated chronic glucose and SFA (e.g., palmitic acid) concentrations on specific organs (e.g., the pancreas), micro-organs (e.g., islets) and cells (e.g., beta cells) [6].

## 2. Systemic and Islet Inflammation in Obesity and Diabetes

Overnutrition, particularly with high SF intake, results in fat accretion, weight gain and obesity [7]. After ingestion, adipose tissue stores ~90% of the non-esterified fatty acid (NEFA) load, and thus substantial adipocyte remodeling occurs. This primarily involves adipose tissue hypertrophy with some hyperplasia to meet triglyceride storage requirements [7]. Hypertrophic adipocytes have a blunted response to insulin and progressively become more lipolytic thereby releasing an excess of NEFA [7] which contributes to systemic insulin resistance. Systemic insulin resistance manifests when glucose is not sufficiently cleared from circulation for uptake in organs for metabolism and storage. This initiates and exacerbates hyperglycemia. In obesity, inflammation leads to systemic insulin resistance and decreases in beta cell mass thereby contributing to beta cell death, dysfunction, failure, and ultimately diabetes. Thus, in obesity, systemic insulin resistance is exacerbated due to hypertrophic adipocytes that do not effectively respond to glucose uptake which worsens glucotoxicity (i.e., hyperglycemia exacerbates); and with excess NEFA, especially SFA release by lipolysis, lipotoxicity compounds and synergizes glucolipotoxicity. In overnutrition and obesity, adiposopathy refers to the response of adipose tissue, behavioral changes and environmental factors [8] characterized by a shift to visceral adipose tissue distribution, a pro-inflammatory imbalance, and ectopic fat deposition when storage capacity exceeds the threshold [9] e.g., in the pancreas. Type 2 diabetic patients had elevated pancreatic triglyceride content [10,11,12,13]. Furthermore, pancreatic fat accumulation induced insulin resistance [13] and impaired insulin secretion [14]. Therefore, intra-pancreatic lipids that are lipotoxic, coupled to hyperglycemia that prevails in diabetes, which is glucotoxic, synergize as glucolipotoxicity thereby worsening diabetic outcomes.

An increase in pro-inflammatory mediator gene expression supports the elevated production of cytokines, chemokines, and other pro-inflammatory mediators, such as 12-lipoxygenase (12LO) [7]. In adipose tissue, 12LO recruits and activates immune cells, such as macrophages (M1), natural killer cells (NK), T cells (CD4^+^ and CD8^+^) and dendritic cells [7]. Immune cells that infiltrate adipose tissue are pro-inflammatory mediators [7]. Thus, adipose tissue insulin resistance manifests. Various adipokines modulate increased leptin and reduced adiponectin production [7]. Hyperleptinemia and leptin resistance are implicated in obesity, inflammation, and impaired beta cell function. These circulating pro-inflammatory mediators induce beta cell dysfunction [7] characterized by impaired glucose-stimulated insulin secretion (GSIS). In beta cells, chronic high NEFA (particularly SFA) concentrations prompt lipotoxicity, endoplasmic reticulum (ER) and oxidative stress thereby leading to mitochondrial dysfunction [7]. Hyperleptinemia concomitant with beta cell leptin resistance likely impairs GSIS [7] with GSIS further impaired by pro-inflammatory mediators and the infiltrating immune cells in adipose tissue and islets [7]. In obesity and glucolipotoxicity, there is immune cell migration, infiltration and amplification in beta cells which induces and exacerbates beta cell inflammation thereby leading to beta cell demise.

In obesity, chronic inflammation primarily involves mononuclear cells (without an acute immunovascular phase) [15] with a typical 2–3-fold non-site specific increase in pro-inflammatory cytokines and chemokines, i.e., not site specific but manifested in various organs e.g., the eye, kidney, heart, liver, adipose tissue and pancreas [15]. Nuclear factor of kappa light polypeptide gene enhancer in B cells (NFκB) regulates NEFA-induced beta cell inflammation and death [16]. In islets, innate immune system activation, characterized by an increase in innate immune cells and pro-inflammatory mediators, impairs beta cell mass and function [17,18]. Obesity and inflammation (i.e., chemokine and cytokine secretion) impair GSIS [19,20,21]. Obesity changes the cellular fate of islet macrophages, i.e., reprograms islet macrophages, to acquire specialized functions e.g., synapse formation and cell-cell adhesion [21]. These pathways are activated for macrophage-beta cell interactions [21], which to some extent, explains the transference of vesicles containing insulin from beta cells to macrophages, which is greatly augmented in obesity [21].

The islet macrophages comprise the intra-islet CD11c^+^ cells (associated with obesity) and peri-islet CD11c^−^ cells, and, in obesity, myeloid lineage cells dominate islet inflammation [22]. As obesity progresses with weight gain with age, in parallel to chronic inflammation characterized by elevated pro-inflammatory cytokines and chemokines, compensatory mechanisms are induced that shift the homeostatic thresholds, which leads to the onset of diabetes [23]. Inflammation triggers an increase in macrophage migration and infiltration in peripheral organs prompting cellular and organ dysfunction; in beta cells, GSIS is impaired [24,25,26]. Islet and beta cells can initiate islet inflammation through sensing stimuli and secreting cytokines, chemokines and islet amyloid peptide (IAPP) to activate macrophages. Diabetes is a chronic inflammatory disease and typically islet cell inflammation can be induced in response to systemic inflammation and other stimuli. This increases the complexity in the prevention, pathogenesis and treatment of diabetes, and the preceding pathologies viz. obesity, insulin resistance, glucose intolerance, and beta cell death, dysfunction, and failure.

## 3. Mediators of Islet Inflammation

Interleukin 1 (IL1) signaling is integral for beta cell physiology and inflammation. Islets richly express the IL1 receptor type I (IL1R) and beta cells are sensitive to its ligands, IL1α and IL1β (a major regulator of inflammation), therefore IL1R signaling regulates beta cell health and physiology [27]. In islets, IL1β secretion occurs due to elevated glucose, thereby initiating inflammation by recruiting and activating macrophages to sustain islet inflammation [28]. The activation of the Nod-like receptor family pyrin domain-containing protein 3 (NLRP3) inflammasome, which produces mature IL1β, was demonstrated in the pancreas and responds to various stimuli [29]. The NLRP3 inflammasome is a critical sensor of nutrient overload, which processes pro-IL1β to its active ILβ in metabolic diseases [30]. The deletion of the NLRP3 inflammasome improves beta cell physiology and viability during oxidative stress and hypoxia that may be associated with anti-inflammatory effects, such as attenuated macrophage islet infiltration [31]. Tumor necrosis factor alpha (TNFα), IL6 and C-X-C motif chemokine ligand 1 (CXCL1) may have additive effects on IL1β [30].

IL1β and insulin have a complex relationship. IL1β and insulin promote each other [30] and have potent effects on glucose homeostasis and inflammation, supporting their roles in the physiology and pathology of metabolism [30]. In macrophages, IL1β and insulin increased glucose uptake, and insulin reinforced inflammation through insulin receptor signaling, glucose metabolism, reactive oxygen species (ROS) production, and NLRP3 inflammasome-mediated IL1β secretion [30]. Insulin enhances IL1β production [30], and up-regulates insulin receptors, phosphatidylinositol 3-kinase-protein kinase B (PI3K-Akt) signaling, glucose transporter 1 (GLUT1)-mediated glucose uptake, glucose metabolism, and ROS generation to activate the NLRP3 inflammasome [32,33]. Furthermore, insulin and IL1β stimulate glucose uptake in muscle and fat (for glucose disposal), and in immune cells (to fuel the immune system) [30].

IL antagonism presents an attractive strategy for beta cell protection and preservation. In macrophages and islets, blocking IL1 signaling with an IL1R antagonist (IL1Ra) limited IAPP-induced secretion of pro-inflammatory cytokines [34], suggesting that IL1 was required for islet inflammation in IAPP formation [35]. A cell cycle regulatory factor, p27^kip1^ (p27), plays a role in the proliferation of inflammatory cells as p27^−/−^ mice had severe functional islet injury, with increased circulating IL1 and TNFα levels that induced macrophage proliferation [36]. In beta cells, another cell cycle regulator and transcription factor, E2F1, regulated insulin secretion through the Kir6.2 promoter [37,38]. E2F1 overexpression partially prevented IL1β-mediated inhibition of Kir6.2 expression and on GSIS [39]. In mice with beta cell IL1Ra knockout, the reduction in proliferation genes resulted in impaired beta cell proliferation and function, through the E2F1-Kir6.2 pathway; thus the benefits of IL1 antagonism appear to be related to beta cell turnover and function [39] and may provide insights in the treatment of diabetes.

Beta cell microRNAs (miRs) are also implicated in islet inflammation and associated with IL signaling [40,41,42]. Pro-inflammatory mediators induced miR-146a-5p expression in human islets, MIN6 mouse beta cells and INS1 rat beta cells [41,43,44], to adapt to IL1β-mediated NFκB activation (as there are NFκB binding elements on the miR-146a promoter [41,45,46]). In INS1 cells transfected with miR-146a-5p, there was reduced NFκB and inducible nitric oxide synthase (iNOS) promotor activity that subsequently decreased cytokine-mediated iNOS protein expression, nitic oxide (NO) synthesis and mitogen-activated protein kinase (MAPK) signaling [44]. Therefore, miR-146a-5p down-regulated islet inflammation and beta cell death by impairing NFκB and MAPK signaling [44].

In the inflamed beta cells of hyperglycemic non-obese diabetic mice, miR-203a (which was up-regulated) targeted insulin receptor substrate 2 (IRS2) thereby regulating beta cell proliferation and apoptosis [47]. In MIN6 cells, IRS2 overexpression may protect from the reduced proliferation and apoptosis induced by miR-203a [47]. Furthermore, MIN6 cells transfected with Irs2 siRNA antagonized the effects of miR203a inhibitors which suggested that Irs2 and IRS2 may be novel targets of miR-203a; and miR-203a inhibitors and IRS2 may be novel therapies [47].

## 4. Metabolic Interplay of Islet Inflammation, Glucolipotoxicity, and Beta Cell Dysfunction

In diabetes, several factors drive a decline in beta cell mass including cellular stress due to compensatory insulin overproduction [48], glucotoxicity due to chronic hyperglycemia, lipotoxicity due to chronic high dietary SFA consumption [49], glucolipotoxicity, inflammation [32], autoimmunity [50] and obesity [3]. However, there are interrelations which require unravelling. High dietary SFA intake contributes to glucotoxicity. Prolonged SFA exposure induces lipotoxicity that diminishes beta cell mass and function, thereby contributing to and exacerbating hyperglycemia. Persistent lipotoxicity thus exacerbates beta cell glucotoxicity i.e., diminished beta cell mass and function will inadequately respond to elevations in glycemia and hyperglycemia will ensue. Persistent hyperglycemia will further augment glucotoxicity for beta cells. In beta cells, glucotoxicity and resident pro-inflammatory macrophages contribute to increased IL1β levels and subsequently impaired GSIS [28]. In addition, glucolipotoxicity, with its synergistic deleterious effects, will have even worse outcomes for beta cells.

Moreover, obesity is entwined with inflammation and characterized by compensatory hyperinsulinemia. Overburdening the beta cells, e.g., in hyperinsulinemia, results in the emergence of cellular stress evident by ER and oxidative stress. Cellular stress is accompanied by islet inflammation that further exacerbates islet and beta cell demise; in addition, autoimmunity is a trait of compromised beta cells in type 1 diabetes that prompts beta cell death. The obese-inflammatory metabolic state is fueled by aberrant cytokine generation, increased synthesis of acute-phase reactants (such as C-reactive protein (CRP)), and an activated pro-inflammatory response [51]. In adults, latent autoimmune diabetes (LADA) is characterized by mild autoimmunity with gradual progression to insulin dependence (relative to type 1 diabetes), concomitant with some type 2 diabetes features such as insulin resistance [52] and overweight/obesity [53]. These interrelations reflect the complexity in beta cell mechanisms and dynamics as they adapt (i.e., compensate) to maintain function in response to variable metabolic demands.

In murine and human islets, ER stress can also be induced by palmitate [54]. The beta cell can itself activate islet inflammation as resident macrophages sense beta cell activity by reacting to ATP, which is co-secreted with insulin, thereby activating macrophages and resulting in inflammation [55]. Beta cell self-activation of islet inflammation is an interesting phenomenon that requires more investigation into what triggers the process, and further delineation of islet inflammation and autoimmunity. This may provide insights on beta cell dedifferentiation to escape attack and death.

## 5. Beta Cell Dysfunctional Transitions

### 5.1. Beta Cell Physiology (Optimal Beta Cell Function)

Beta cells require normal beta cell integrity, i.e., number, size, and machinery, to effectively respond to constantly fluctuating metabolic demand for insulin [56] by rapidly equilibrating glucose across the plasma membrane for insulin exocytosis [57]. This requires adequate insulin biosynthesis and maintaining beta cell readiness for GSIS while regulating glycemia within a tight physiological range; and depends on the coordinated regulation of insulin secretion through nutrient availability, hormonal and neural inputs [58]. As the key regulator of beta cell physiology, glucose coordinates and stimulates insulin gene transcription, pro/insulin biosynthesis and protein translation, and insulin secretion [59] and maintains the highly differentiated and specialized beta cells to meet insulin biosynthesis and secretory demands [60].

Furthermore, beta cells are equipped with highly developed and active ER, for their role in folding, export, and processing of newly biosynthesized insulin [61]. Maintaining optimal beta cell physiology for insulin biosynthesis and secretion requires efficiently functioning unfolded protein response (UPR) machinery [57]. In beta cells, nuclear factor erythroid 2p45-related factor 2 (Nrf2) regulates many antioxidant defense factors [62]. ROS are necessary for beta cell signaling but excessive ROS induces oxidative stress. Physiologically, in response to Nrf2 activators, GSIS was impaired with altered ROS handling [62]. Nrf2 activators may shield beta cells from glucolipotoxicity, through the preservation of mitochondrial function and redox balance, to maintain GSIS [62].

**Figure 1:**
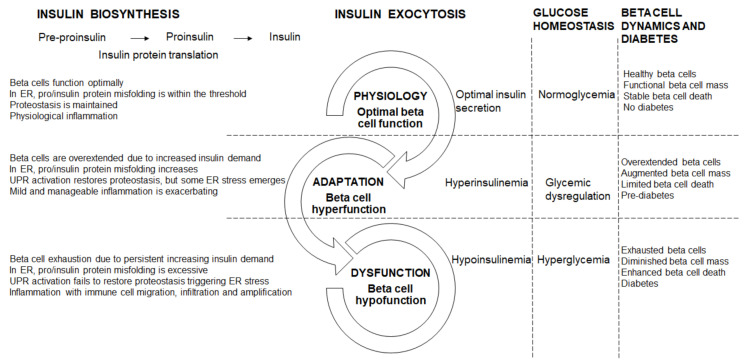
Beta cell physiology and dysfunctional transitions.

The IL1R signaling system maintains beta cell physiology—with pancreatic IL1R knockout, in lean mice, whole body glucose homeostasis was disrupted which was exacerbated in obese db/db mice, concomitant with reduced insulin content and GSIS, both in vivo and ex vivo [27]. Furthermore, in mouse and human islets ex vivo, IL1R signaling enhanced the docking of insulin granules, supporting a role for IL1R activation in refining beta cell physiology [27,63]. Optimal beta cell physiology is therefore characterized by sufficient insulin biosynthesis and secretion; in the ER, pro/insulin protein misfolding is well within the threshold and therefore proteostasis is maintained; with inflammation in the physiological range (Figure 1). Healthy beta cells maintain their function through adequate beta cell mass, and stable beta cell death, which prevents diabetes by keeping metabolism and physiology intact (Figure 1).

### 5.2. Beta Cell Hyperfunction

Physiologically, healthy beta cells respond to the nutritional cues with biphasic insulin secretion: the first phase needs a fast and substantial increase in intracellular calcium concentrations to release insulin granules from a limited pool, whereas the second phase also requires an increase in intracellular calcium concentrations but is amplified by glucose to replenish the pool of releasable granules [64]. However, in overnutrition, there is excessive nutrient secretagogues e.g., glucose, lipids, and amino acids that initially induces mild nutritional metabolic stress on the beta cells, thereby elevating basal and enhancing amplification of insulin secretion, resulting in hyperinsulinemia [57] that reflects hyperfunction. During pre-diabetes and early diabetes, beta cells adapt in response to muscle, hepatic and adipose tissue insulin resistance by enhancing GSIS [62] and their hyperfunction largely contributes to increased insulin output, which is supported by beta cell mass expansion [65]. Beta cell mass expands by hyperplasia through increased replication and neogenesis from the recruitment of progenitors, and hypertrophy to establish and sustain hyperinsulinemia [66] thereby restoring glycemia. In insulin resistant and impaired glucose tolerant individuals, beta cell mass is primarily replenished through neogenesis [67,68]. Beta cell hyperplasia, concomitant with beta cell hypertrophy, initially support beta cell adaptation to restore glucose homeostasis through increased insulin biosynthesis and secretion to meet greater demand.

Often beta cell hyperfunction has adverse consequences. In db/db mice [69], ZDF rats [24,70], and obese humans [63,71], the up-regulation of the IL1R ligands supports the adaptive response to sustain insulin biosynthesis and secretion [27]. In early diabetes, an increase in insulin may drive and sustain inflammation in macrophages and thereby contribute to the chronic low-grade inflammation [30]. In beta cell hyperfunction, protein misfolding starts to breach the cellular threshold as ER start to overload. Beta cells become overextended due to the progressively increasing insulin demand (Figure 1). Furthermore, in ER, pro/insulin protein misfolding increases but remains within the threshold, and although the UPR activation restores some proteostasis, ER stress starts to emerge (Figure 1). Beta cell destruction by the autoimmune infiltration continues and is exacerbated by the increasing metabolic and glycemic overload causing ER and oxidative stress and apoptosis [72]. Physiologically, the pro-apoptotic factors, Bax and Bak, do not influence glucose-stimulated Ca^2+^ responses, insulin secretion or glucose tolerance [73]. However, unresolved ER stress triggered Bax and Bak-dependent death in murine fibroblasts [74,75,76,77]; in islets, during early glucolipotoxicity-induced ER stress, Bax and Bak deletion amplified the UPR [73]. Oxidative stress is also induced during beta cell hyperfunction. The pro-inflammatory response is mild and manageable. Hyperinsulinemia helps to maintain some a level of normoglycemia, through a greater functional load per beta cell, with glycemia steadily increasing towards glucose dysregulation (Figure 1). During hyperfunction in pre-diabetes, the functionally overextended beta cells are supported by enhanced mass, with beta cell death playing a limited role (Figure 1).

### 5.3. Beta Cell Hypofunction

When the beta cell adaptation fails, hyperglycemia and diabetes develop due to insulin deficiency [65]. The shift from beta cell hyperfunction to hypofunction is driven by increasing severity in hyperglycemia, hyperlipidemia, and glucolipotoxicity. In beta cells, ROS/reactive nitrogen species (RNS) production can exacerbate ER stress and induce cell death [78] that progressively diminishes beta cell function [66]. Islet inflammation is attributed to overnutrition, e.g., glucolipotoxicity, that leads to beta cell exhaustion, cytokine and chemokine synthesis, that triggers immune cells recruitment to islets and beta cells [79] to further amplify beta cell dysfunction and insulin resistance [56]. Increased protein misfolding and ER overloading leading to ER stress, concomitant with oxidative stress (as increases in ROS/RNS are not sufficiently neutralized by antioxidant enzymes) and inflammation all converge towards further deteriorating beta cell dysfunction. Chronic IL1 exposure contributed to beta cell death and dysfunction by altering gene transcription, protein activity and inducing oxidative stress [80]. As diabetes progresses, increased IL1β expression was linked to insulin resistance and beta cell destruction [19]; with damaged beta cells eventually failing to respond to increasing insulin demand [62], due their rapid death and functional impairment [65]. As beta cells are overextended towards exhaustion, they hypofunction characterized by hypoplasia, hypotrophy, reduced proliferation and neogenesis and dedifferentiation [66] as they lose their specialized function and identity. Thus, the highly differentiated and specialized state of beta cells is eroded, and they are reduced in number and size (through beta cell death and non-replenishment from progenitors), which renders a sub-critical mass of residual beta cells to maintain their physiology. Beta cell dedifferentiation also contributes to reduced beta cell mass and a marked reduction in beta cell mass presents closer to overt diabetes [65]. As beta cell dysfunction further deteriorates, beta cells cannot meet the persistent demand for increased insulin biosynthesis and secretion in response to persevering elevated glycemia i.e., chronic hyperglycemia. Prolonged chronic hyperglycemia triggers beta cell exhaustion resulting in hypofunction, characterized by hypoinsulinemia which is insufficiently responsive to increases in glycemia. In overt beta cell dysfunction, when beta cells are exhausted towards failure, there is reduced insulin biosynthesis and insufficient insulin secretion [66]—i.e., more insulin is required, e.g., exogenously to maintain glucose homeostasis—and there is decreased insulin signaling in peripheral organs i.e., reduced glucose uptake, with prevailing hyperglycemia that progressively exacerbates.

Beta cells that are continuously overextended by persistently increased insulin demand become exhausted (Figure 1). In ER, pro/insulin protein misfolding increases beyond the threshold, and with the ER overload, the UPR is activated but does not restore proteostasis, therefore ER stress manifests and is exacerbated by oxidative stress (Figure 1). Islet and beta cell pro-inflammatory responses are triggered with immune cell migration, infiltration and amplification (Figure 1). Inflammatory pathways are a complex, intertwined, cascading sequence of events that converge to induce beta cell death, and diminish beta cell mass [7] and function leading to beta cell failure and diabetes. In diabetes, in inflamed islets, the islet macrophage inflammasome-IL1β pathway is a common pathway for beta cell dysfunction [81]. In rodent diabetes, islet inflammation and macrophage infiltration induce beta cell failure [81]. Chronic islet and beta cell glucolipotoxicity induces and exacerbates beta cell dysfunction and death, and accelerates beta cell metabolic overload, thereby impairing beta cell adaptation that progresses towards beta cell failure [82]. During hypofunction, beta cells become exhausted as increased beta cell death contributes to reduced beta cell mass (Figure 1). Fatigued beta cells fail and diabetes manifests.

### 5.4. Beta Cell Failure and Diabetes

Beta cell dysfunctional transitions are initiated with hyperfunction that precedes hypofunction and advances to beta cell failure. Beta cell death occurs in healthy beta cells, but with the dysfunctional transitions i.e., from hyper- to hypofunction to failure, the beta cell population progressively diminishes through increased beta cell death. Although beta cells are resilient and will compensate to cope with insulin demand despite reduced numbers [56], with persistent hyperglycemia, there is increased beta cell death, and with less beta cells available to function (i.e., inadequate beta cell capacity), they are therefore overburdened towards exhaustion, beta cell failure manifests, and diabetes ensues.

## 6. Conclusions

Beta cells serve to prevent metabolic diseases by biosynthesizing and secreting insulin to maintain glucose homeostasis. Stressed and inflamed beta cells are functionally compromised and cannot adapt to effectively respond to increased insulin demand which advances beta cell dysfunction towards failure and diabetes. Beta cell preservation and replenishment, through novel agents, healthy lifestyles of balanced diets and regular exercise, maintains beta cell physiology and sustains adaptation [56]. Novel therapies should focus on beta cell protection and functional recovery in early diabetes, and support beta cell mass replacement in late diabetes [65]. Targeting pro-inflammatory mediators and redox balance are viable strategies for diabetes prevention and treatment, given their roles in the beta cell dysfunctional transitions.
